# PROTEIN INTAKE AND MORTALITY IN OLDER ADULTS WITH CHRONIC KIDNEY DISEASE: A MULTICOHORT POPULATION-BASED STUDY

**DOI:** 10.1093/geroni/igae098.1456

**Published:** 2024-12-31

**Authors:** Adrián Carballo-Casla, Carla Maria Avesani, Giorgi Beridze, Rosario Ortolá, Juan-Jesús Carrero, Fernando Rodríguez-Artalejo, Davide Liborio Vetrano, Amaia Calderón-Larrañaga

**Affiliations:** Karolinska Institutet, Stockholm, Stockholms Lan, Sweden; Karolinska Institutet, Stockholm, Stockholms Lan, Sweden; Karolinska Institutet, Stockholm, Stockholms Lan, Sweden; Autonomous University of Madrid, Madrid, Madrid, Spain; Karolinska Institutet, Stockholm, Stockholms Lan, Sweden; Autonomous University of Madrid, Madrid, La Rioja, Spain; Karolinska Institutet, Stockholm, Stockholms Lan, Sweden; Karolinska Institutet, Stockholm, Stockholms Lan, Sweden

## Abstract

Avoiding high protein intake in older adults with chronic kidney disease (CKD) may reduce the risk of kidney function decline, but associations with nutritional status and survival are not well known. We aimed to study the associations of total, animal, and plant protein intake with all-cause mortality in older adults without and with mild or moderate CKD. We used data from three cohorts (Seniors-ENRICA 1 and Seniors-ENRICA 2 in Spain, and SNAC-K in Sweden) comprising 8,539 community-dwelling adults ≥60 years. CKD was ascertained according to estimated glomerular filtration rates, urine albumin excretion, and diagnoses from medical records. Cumulative protein intake was estimated via validated dietary histories and food frequency questionnaires. Vital status was ascertained with national death registers. Associations were estimated with Cox proportional hazards regression models, adjusted for sociodemographic, lifestyle, morbidity, and dietary variables. After a maximum follow-up of 15 years, 1,901 deaths were recorded. Higher total protein intake was associated with lower mortality among participants with CKD [hazard ratio (95% confidence interval) for 1.0, 1.2, and 1.4 g/kg/day versus 0.8 g/kg/day = 0.90 (0.83,0.98), 0.82 (0.70,0.96), and 0.75 (0.62,0.92), respectively]. Plant protein showed a stronger inverse association than animal protein [0.77 (0.64,0.92) and 0.92 (0.86,0.98) per 0.2 g/kg/day increment, respectively]. Among participants without CKD, most associations were stronger, but no statistically significant interactions between protein intake and CKD arose. Higher protein intake, particularly plant protein, was associated with lower mortality in older adults with mild or moderate CKD. Associations did not differ substantially in older persons without CKD.

